# Clinical characterization and factors associated with quality of life in Long COVID patients: Secondary data analysis from a randomized clinical trial

**DOI:** 10.1371/journal.pone.0278728

**Published:** 2023-05-16

**Authors:** Mario Samper-Pardo, Sandra León-Herrera, Bárbara Oliván-Blázquez, Santiago Gascón-Santos, Raquel Sánchez-Recio

**Affiliations:** 1 Institute for Health Research Aragon (IISAragon), Zaragoza, Spain; 2 Department of Psychology and Sociology, University of Zaragoza, Zaragoza, Spain; 3 Network for Research on Chronicity, Primary Care, and Health Promotion (RICAPPS), Barcelona, Spain; 4 Department of Preventive Medicine and Public Health, University of Zaragoza, Zaragoza, Spain; Complutense University of Madrid: Universidad Complutense de Madrid, SPAIN

## Abstract

**Background:**

Long COVID patients suffer a negative impact on their quality of life, as well as their functioning, productivity or socialization. There is a need to better understand the individual experience and circumstances surrounding these patients.

**Objective:**

To characterize clinical picture of Long COVID patients and to identify factors associated with quality of life.

**Methods:**

A secondary data analysis from a randomized clinical trial (RCT) was carried out with 100 Long COVID patients treated by Primary Health Care and residents in the territory of Aragon (northeast of Spain). The main variable of the study was quality of life, evaluated using the SF-36 Questionnaire, in relation to socio-demographic and clinical variables. In addition, ten validated scales were used that contemplated their cognitive, affective, functional and social status, as well as personal constructs. Correlation statistics and linear regression model were calculated.

**Results:**

Long COVID patients suffer a decrease in their levels of physical and mental health. On the one hand, the higher number of persistent symptoms (b = -0.900, p = 0.008), worse physical functioning (b = 1.587, p = 0.002) and sleep quality (b = -0.538, p = 0.035) are predictors of worse quality of life, physical subscale. On the other hand, higher educational level (b = 13.167, p = 0.017), lower number of persistent symptoms (b = -0.621, p = 0.057) and higher affective affectation (b = -1.402, p<0.001) are predictors of worse quality of life, mental subscale.

**Conclusion:**

It is necessary to design rehabilitation programs that consider both the physical and mental health of these patients, thus obtaining an improvement in their quality of life.

## Introduction

Since the World Health Organization (WHO) declared COVID-19 a highly contagious and harmful pandemic for the human species on March 11, 2020 [1–4], the main health organizations around the world, together with government agencies and scientific health corporations, are under the arduous task of expanding scientific knowledge about the pathophysiology of this new coronavirus, thus trying to minimize its spread and consequent impact on quality of life of the world population [5].

Even though most people infected with COVID-19 turn out to be asymptomatic or develop mild-moderate symptoms, it is estimated that around 15% of those affected have progressed to greater severity, requiring hospital care in some cases [6, 7]. Initially, a mean recovery time was established after COVID-19 infection of 2 to 3 weeks until the disappearance of symptoms (except for the recovery of smell and taste, in the case of partial or total alteration) [8–11]. Subsequently, scientific evidence has estimated that a large percentage of those affected could maintain symptoms for 5 weeks or more after the acute infection, and that around 10–20% would see symptoms persist after 12 weeks or more [12]. Given this confusing panorama, the National Institute for Health and Care Excellence (NICE) established that the symptoms of COVID-19 can last 4–12 weeks and people who maintain or develop symptoms for a longer period and that cannot be explained by an alternative diagnosis are considered to be “Post-COVID Syndrome” [13]. In the scientific field, this new disease has been defined in a pragmatic way, "such that after the COVID-19 infection it does not recover after several months", identifying it as "Long COVID" [14, 15]. Given the need to establish the limits of the disease, in October 2021, the WHO registered the definition of this condition as the condition that occurs in adult individuals with a history of probable or confirmed infection by SARS-CoV-2, with symptoms typical of the disease usually 3 months after onset, without explanation by alternative diagnosis, referring to it as "Post COVID Condition" [16].

Despite rapid characterization of the acute phase of COVID-19, the underlying etiology of prolonged symptoms is still limited. The prevalence of Long COVID illness is higher in women (80%) than in men (20%) [17]. The type of symptoms, duration and degree of severity, as well as associated risk factors, are still being studied. It is worth mentioning the international cohort carried out by Davis et al. (2021) has come to count 203 symptoms of this disease [18], coming to involve at least 10 organ systems, as verified by other studies [19]. Among the most predominant symptoms are profound fatigue, myalgia, dyspnea, cough, fever, low-grade fever, dysthermia, palpitations, headache, arthralgia, odynophagia, dizziness, hypotension, bruising and skin rashes, neurological symptoms such as tingling, cognitive deficits, sleep disorders and mental health problems, of the anxious-depressive type [20–27].

The development and evolution of these persistent symptoms supposes a total alteration of the organism, as well as a general malaise characterized mainly by chronic fatigue and musculoskeletal pain [18]. Consequently, there is a great impact on the functioning of the respiratory system, in addition to increasing the potential to develop metabolic, cardiovascular, gastrointestinal, and neurological disorders, among others, and alter the emotional well-being of these patients [28].

The physical and mental repercussions are affecting different areas of life such as family, work or social and consequently, their quality of life [29]. Therefore, Long COVID must be recognized as a condition with disabling potential, at least temporarily [30]. Several investigations report that at least 50% of Long Covid patients continue to see negative effects on their daily activities, which were previously carried out regularly, after 2–5 months and 15% after 8 months of infection [23, 31, 32]. These limitations may occur in activities of daily life such as bathing, dressing, or walking [33]. Additionally, because of cognitive dysfunction and other symptoms, it is estimated that work capacity and personal productivity are lower than pre-disease levels [18].

On the other hand, the persistent symptoms and sequelae add to the psychosocial impact of interrupted access to health care (such as arranging for regular medication), basic personal routines (such as walking to local stores), social interactions (such as meeting with friends) and support networks [34–37]. Therefore, support should be personalized with input from a multi-professional team (e.g., primary care physician, social worker, rehabilitation teams) [38].

This global impact of prolonged COVID should not be ignored. The quality of life of these patients has suffered a great impact. There is an urgent need to offer rehabilitation treatments to long-term COVID patients, as well as help healthcare workers understand what is required for recovery. The health system, research institutes, and public policies should be involved in their response. To do this, it is necessary to better understand the clinical characterization and individual experiences of patients. In summary, the loss of quality of life, the need for health care, as well as the clinical characterization and diagnostic recognition, make this disease an idea worthy of investigation for the health sector [39].

Hence, the objective of this study is to characterize clinical picture of patients diagnosed with Post COVID-19 Syndrome, in relation to sociodemographic, clinical, affective, cognitive, functional and social variables; as well as to identify factors associated with quality of life of these patients from PHC.

## Methodology

### Study design

This research study is a secondary data analysis [40] of data collected at the start of a randomized clinical trial (RCT) [41] called: “Analysis of symptoms and quality of life of people with prolonged diagnosis of COVID-19, and the efficacy of an intervention in primary health care using ICT”, registered on 10/02/2022, with reference number ISRCTN91104012.

### Sample size

The sample size was established in the RCT study, as can be seen in its protocol article [41]. The methodology of the RCT established a necessary sample size of 78 subjects, according to its main variable, "quality of life", through the SF-36 questionnaire. Finally, a total of 100 Long COVID participants were included in this study Therefore, the necessary sample size was exceeded. Of these participants, 20 were men and 80 women.

### Recruitment and participants

The study population has been Post COVID-19 Syndrome patients, of legal age (18 years or older) and treated by Primary Health Care. The exclusion criteria put forward for its collection have been: not having a positive diagnostic test for COVID-19 for more than the previous 3 months; have a diagnosis of severe uncontrolled disease; significant risk of suicide; pregnancy and lactation; participation in a clinical trial in the last six months; existing structured rehabilitative or psychotherapeutic treatment by health professionals and the presence of any medical, psychological or social problem that may significantly interfere with the patient’s participation in the study.

Patients were recruited by PHC professionals, who participated in the clinical trial within a PHC setting, as detailed in the RCT protocol article [41]. The Long COVID Association of Aragon (Spain) also participated in the recruitment.

This process was carried out consecutively until reaching the sample size.

### Variables and instruments

This study contemplated multiple variables that allow us to know about the Long COVID patient from a broad perspective. For this, in addition to sociodemographic and clinical variables, a total of 10 scales were selected.

- Socio-demographic variables: gender (man, woman, other), age, civil status (married or in couple/single, separated, divorced or widowed), education (no studies or primary studies/ secondary or university studies) and occupation (employee, unemployed, employee with temporary work disability (TWD), retired, others).
- Clinical variables related to post-COVID-19: time since infection (months), number of residual symptoms and their severity measured through a Visual Analogue Scale. Persistent symptoms included were: Gastrointestinal symptoms, total or partial loss of smell or taste, eye problems (blurred vision, increased dioptres, dry eyes, conjunctivitis), tiredness or fatigue, cough, sore throat, dyspnea, fever (over 38°C), low-grade fever (37°C—38°C), chills or chills without fever, headaches, drowsiness, dizziness, tachycardia, orthostatic hypotension, bruising, myalgia, joint pain, chest pain, back pain (cervical, dorsal or lumbar), neurological symptoms (tingling, spasms, etc.), cognitive (memory loss, brain fog or confusion or poor attention and concentration capacity), loss of libido or erectile dysfunction, alteration of the menstrual cycle, urinary symptoms (infections, overactive bladder), hair loss and other symptoms that can be considered residual [20–27].
- Quality of life was evaluated by the SF-36 Questionnaire [42], which measures eight dimensions of health: physical function, physical role, aches and pains, general health, vitality, social function, emotional role, and mental health. The eight dimensions define two main components of health: physical summary component and mental summary component. The eight scales are scored from 0 to 100, with higher scores indicating better health status. The official Spanish version of the questionnaire was used [43]. The Cronbach’s alpha obtained was 0,841.
- Cognitive status was assessed by the official Spanish version of the Montreal Cognitive Assessment (MoCA) [44], which assesses six cognitive domains (memory, visuospatial ability, executive function, attention, concentration or working memory, language and temporal-spatial orientation). It is about a total score of 30 points and the cut-off point for the detection of mild cognitive impairment is 26 points. Cronbach’s alpha obtained in this study is 0.457.
- Physical functioning was evaluated using the 30-second Sit to Stand Test [45], specifically used to detect respiratory diseases [46]. The test assesses endurance at high power, speed in terms of muscular endurance or strength, by recording the number of times a person can stand up and sit down completely. It has good test-retest reliability (0.84 <R< 0.92).
- Physical activity was assessed using the International Physical Activity Questionnaire-Short Form (IPAQ-SF) [47]. It assesses the levels of habitual physical activity over the preceding seven days. It has seven items and records activity at four levels of intensity: vigorous-intensity activity and moderate-intensity activity (walking and sitting). The official Spanish version was used [48]. The minute walking score was used for the analysis.
- Affective status was assessed through the Hospital Anxiety and Depression Scale (HADS) questionnaire [49]. The HADS is a self-report-based scale that was developed to screen for depression and anxiety disorders in medical patients in primary care settings. The HADS includes 14 items that assess symptoms of anxiety and depression, each item corresponding to a 4-point Likert-type scale (zero to three), with scores ranging from 0 to 42 for its total score. Higher scores indicate more severe symptoms. The HADS has been translated into several languages, including Spanish [50]. Cronbach’s alpha obtained in this study is 0.91.
- Sleep quality was assessed using the Insomnia Severity Index questionnaire (ISI). The ISI [51] measures the patient’s perception of nocturnal and daytime symptoms of insomnia. This self-report scale has seven items, with each response ranging from zero to four, and an overall score ranging from 0 to 28, with a higher score indicating greater severity of insomnia. The Spanish version was used (Cronbach’s alpha = 0.82) [52]. In this study, the Cronbach’s alpha obtained was 0.86.
- Social Support was evaluated using the Medical Outcomes Study Social Support Survey (MOS-SS) [53]. It is a self-report instrument consisting of four subscales (emotional/informational, tangible, affectionate and positive social interaction) and an overall functional social support index. It has 19 items and uses a 5-point Likert Scale. Higher scores indicate more support. The official Spanish version was used (Cronbach’s alpha ≥0.91) [54]. Cronbach’s alpha obtained in this study was 0.94.
- Personal constructs. The personal factors relating to behaviour that were collected are the following:
  - Self-efficacy was evaluated using the Self-Efficacy Scale-12 (GSES-12). This scale has 3 factors: Initiative (willingness to initiate the behavior), Effort (willingness to try to complete the behavior), and Persistence (persevering to complete the task in the face of adversity). The official scale obtained a Cronbach’s alpha of 0.69 [55]. Cronbach’s alpha obtained in this study was 0.76.
  - Patient activation in their own health was evaluated using the Patient Activation Measure (PAM) questionnaire regarding the management of their health [56]. It evaluates the patient’s perceived knowledge, skills and confidence to engage in self-management activities through 13 items with a Likert Scale from one (strongly disagree) to four (strongly agree). The resulting score ranges between 13 and 52. Higher scores indicate higher levels of activation. The official Spanish version for chronically ill patients was used (Cronbach’s alpha = 0.98) [57]. The Cronbach’s alpha obtained in this study was 0.87.
  - Health literacy was evaluated using the Health Literacy Europe Questionnaire (HLS-EUQ16) [58]. Health literacy is defined as the population’s knowledge, motivation, and individual capacity to understand and make decisions related to promoting and maintaining their health. It contains 16 items, ranging from 1 to 4. Higher scores indicate worse health literacy. The official Spanish version for chronically ill patients was used (Cronbach’s alpha = 0.98) [59]. The Cronbach’s alpha obtained in this study was 0.87.

### Statistical analysis

Statistical analyses were carried out using the IBM SPSS Statistics version 22.0.0.0 and Microsoft Excel computer programs. First, the sample distribution was analyzed, obtaining Shapiro–Wilk statistic values that were lower than 0.05 for all of the variables except for the number of symptoms, SF-36 general health and SF-36 mental health. However, non-parametric statistics were used. Subsequently, a descriptive analysis was performed: in cases of quantitative variables, median, mean, standard deviation and interquartile range were used; frequency and percentages were used for qualitative variables. In addition, a descriptive analysis was carried out according to sex. To verify if there were significant differences, the chi-square test was performed for quantitative variables and for qualitative variables it was performed using the Mann-Whitney U and the Kruskal-Wallis test. A bivariate analysis was performed; SF-36 physical health and SF-36 mental health were analyzed as a continuous scale with a minimum of 0 and a maximum of 100. Spearman correlations between SF-36 physical health or SF-36 mental health and the rest of the continuous variables were calculated. This bivariate analysis for qualitative variables was also performed using Mann-Whitney U and Kruskal-Wallis test, and the chi-square test was performed for quantitative variables. A linear multivariate model was developed for SF-36 physical health and SF-36 mental health as dependent variables. The independent variables (sociodemographic variables, number of persistent symptoms, MoCA, sit to stand, HADS, IPAQ, ISI, MOS, GSES-12, PAM and HLS-EUQ16) were added into the regression model [60], and a final model was obtained. Confounder variables were not adjusted in the linear regression analysis. In the model, occupation was introduced as having or not an active skilled occupation. In addition, a multicollinearity test was performed. Linear regression was used since the residuals of the model had a finite mean, constant variance, and normal distribution. However, bootstrapping analysis with 2000 samples was also conducted. All levels of significance were established at 0.05.

### Ethics considerations

Ethics approval was granted by the Clinical Research Ethics Committee of Aragon (PI21/139 and PI21/454). The procedures carried out for the creation of this work complied with the ethical standards of the previously mentioned committee and with the 1975 Declaration of Helsinki. All of the subjects signed an informed consent form, their data were anonymised and will only be used for the purposes of the study. Participants and healthcare professionals will be informed about the results. The ethics committee will be notified of any protocol modifications.

## Results

A total of 100 people participated, of which 80 were women and 20 men. The median age was 47 years (IQR 11 years, range: 29–72). Table 1 presents the description of the total sample, as well as the comparison by gender, based on the variables collected. The profile of the participant was a woman, whose age was around 48 years old, married, with secondary or university studies, employed or temporarily unable to work, with low quality of life, but high social support and perception of self-efficacy. There are no significant differences by sex in sociodemographic and clinical variables, except for general health assessed by the SF-36, in which women have a significantly higher score than men. The 8 dimensions of quality of life of the SF-36 have been regrouped into 2 variables, the SF-36. 36 Physical Health and SF-36 Mental Health, to obtain a broader view, in which there are no significant differences by gender. Likewise, for both men and women, the median scores on the cognitive assessment (MoCA) and physical functioning (Sit to Stand Find), indicate a deterioration in physical and cognitive functioning. The self-efficacy scales (PAM and HLS-EUQ16) also collect negative scores in Long COVID patients.

**Table 1:** Description of sociodemographic and clinical variables of the total sample and comparing by gender.

Variables	Total sample N (%) mean (SD)/median (IQR)	Male N(%) mean (SD)/median (IQR)	Female N(%) mean (SD)/median (IQR)	p-value
Gender				
Male	20 (20%)			
Female	80 (80%)			
Age	48,28 (9.27)/47 (11)	48 (8.3) / 49.5 (8.75)	48.35 (9.54) / 47 (14)	0.918
Marital status				
Married or in couple	70 (70%)	15 (75%)	55 (68.8%)	0.585
Single, separated, divorced or widowed	30 (30%)	5 (25%)	25 (31.2%)	
Educational level				
Primary studies	9 (9%)	4 (20%)	5 (6.3%)	0.055
Secondary or university studies	91 (91%)	16 (80%)	75 (93.7%)	
Occupation				
Employee	46 (46.9%)	5 (25%)	41 (52.6%)	
Unemployed	5 (5.1%)	0	5 (6.4%)	
TWD	37 (37.8%)	13 (65%)	24 (30.8%)	0.059
Retired	9 (9.2%)	2 (10%)	7 (9%)	
Others	1 (1%)	0	1 (1.2%)	
SF-36				
Physical function	51,05 (25,11) / 50 (40)	58.5 (26.26) / 57.5 (26.26)	49.18 (24.64) / 47,5(24.64)	0.163
Physical role	6.75 (21.86) / 0 (0)	10 (27.38) / (0 (0)	5.93 (20.37) / 0 (0)	0.510
Bodily pain	32.61 (26.03) / 22 (30)	29.6 (23.7) / 22 (22.70)	33.36 (26.67) / 22 (26.67)	0.599
General health	38.35 (17.74) / 40 (25)	30.95 (17.4) /27.5 (17.40)	40.2 (17.44) / 40 (17.44)	0.022
Vitality	27.5 (13.86) / 25 (25)	31 (13.43) / 32.5 (13.43)	26.62 (13.91) / 25 (13.91)	0.172
Social function	39(29.85) / 31.25(46.87)	33.12 (28.75) / 25 (28.75)	40.46 (30.11) / 37.5 (30.11)	0.317
Emotional role	21 (39.54) / 0 (0)	25 (44.42) / 0 (0)	20 (38.46) / 0 (0)	0.795
Mental health	51,6 (15.96) / 52 (24)	49 (17.16) / 44 (36)	52.25 (15.69) / 52 (15.69)	0.294
SF-36 Physical Health	32.19(16.61) /28.5 (20.06)	32.26 (17.77) / 24.88 (19.5)	32.17 (16.43) /29.13 (16.43)	0.701
SF-36 Mental Health	34.77 (19.3) / 29.06(26.16)	34.53 (19.83) / 25.75 (18.59)	34.84 (19.3) / 30.06 (19.3)	0.766
N° persist. symptoms	16.47 (5.99) / 16.5 (8)	13.85 (6.54) /14 (13.25)	17.12 (5.71) / 17 (8.75)	0.058
MoCA	23.64 (3.85) / 25 (4.75)	22.1 (4.67) / 22 (6.25)	24.02 (3.54) / 25 (3)	0.068
Sit to Stand Test	10.37 (3.49) / 10.5 (4)	10 (3.56) / 10 (4)	10.46 (3.49) / 11 (4)	0.621
HADS	17.61 (8.31) / 16 (12)	18.45 (9.98) / 20 (16)	17.4 (7.9) / 16 (11.5)	0.685
ISI	11.34 (6.58) / 11.5 (11)	13.1 (7.13) / 12 (10.5)	10.9 (6.41) / 10 (11.5)	0.229
MOS-SS	83.84 (16.33) / 91 (29)	83.65 (18.42) / 92,5 (18.25)	88.88 (15.89) / 91 (29)	0.692
IPAQ-SF	338.9 (349.24) / 257,5 (288.75)	394.7 (280.7) /297,5 (446,25)	324.93 (364.56) / 240 (315)	0,168
GSES-12	44.66 (7.51) / 46 (10)	43.9 (9.26) / 47,50 (11)	44.85 (7.07) / 46 (8.75)	0,846
PAM	39.82 (6.16) / 40 (8.75)	39.3 (5.57) / 40 (7.5)	39.95 (6.44) / 40 (4.75)	0,710
HLS-EUQ16	32.1 (7.03) / 32.5 (8.75)	32.4 (5.57) / 33 (9.75)	32.02 (7.38) / 32 (9)	0.714

**Fig 1:**
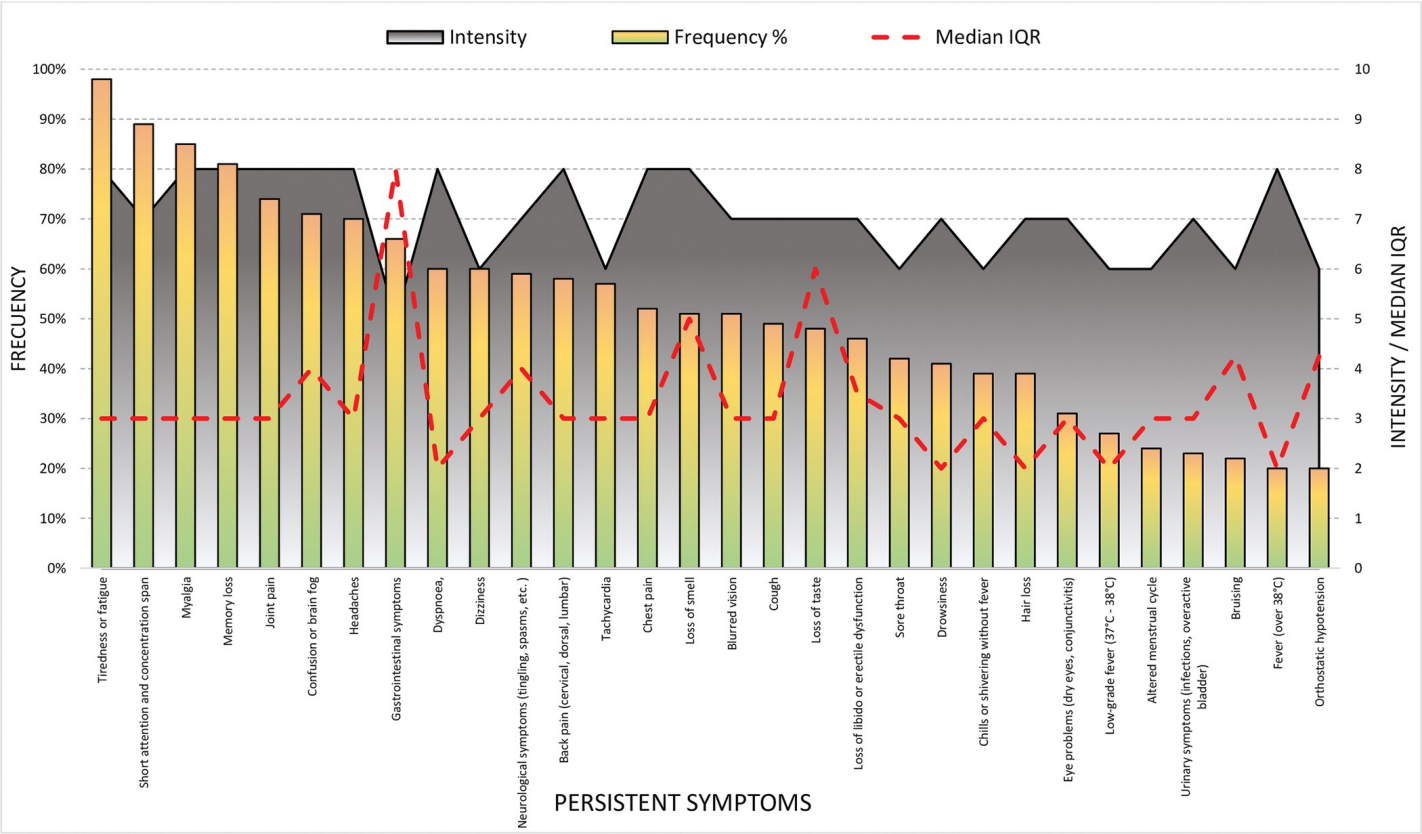
Description of persistent symptomatology, frequency and intensity.

Delving into the persistent symptoms, as can be observed on Fig 1, the median the time since the contagious is 18 months and the number of persistent symptoms is 16.5 (IQR 8). The most frequent symptoms are tiredness or fatigue (98%), myalgia (85%), joint pain (74%), memory loss (81%), confusion or brain fog (71%) and short attention and concentration span (89%), and having an intensity of 7 or 8 points above 10.

Tables 2 and 3 show the bivariant analysis related the quality of life (SF-36 physical health and SF-36 mental health) and the collected variables. There is a relationship between the SF-36 physical health and occupation, the number of persistent symptoms, physical and cognitive functioning, affective state, patient´s activation and health literacy. Higher number of persistent symptoms, cognitive and affective affectation, higher activation and lower health literacy, lower SF-36 physical health score; while higher physical functioning and patient´s activation are associated to a higher SF-36 physical health score. Employees and retired patients have a higher SF-36 physical health score compared to unemployed patients and patients with sick leave. Regarding SF-36 mental health score, there is a relationship between SF-36 mental health score and the number of persistent symptoms, physical and cognitive functioning, affective state, sleep quality, physical activity and self-efficacy. Higher number of persistent symptoms, cognitive and affective affectation, and sleep quality affectation, lower SF-36 mental health score; on the other hand, higher physical functioning, physical activity and self-efficacy are associated to a higher SF-36 mental health score.

**Table 2:** Comparation SF-36 physical health score and SF-36 mental health score, according to the gender, marital status, educational level, and employment status.

Variables	SF-36 Physical health				SF-36 mental health			
	Median (IQR)	P-value	Confidence interval 95%		Median (IQR)	P-value	Confidence interval 95%	
			Inferior	Superior			Inferior	Superior
Gender								
Men	24.87 (20.81)	0.701	-8,500	6,250	25.75 (35.72)	0.776	-9,500	7,500
Women	29.12 (20.56)				30.06 (23.69)			
Marital status								
Married or in couple	27.75 (17.06)	0.746	-5.000	7.500	33,25 (28.25)	0.724	-9.625	6.875
Single, separated, widowed	29 (23.63)				28 (23.47)			
Educational level								
Without studies or primary studies	26.50 (13.88)	0.796	-11,250	7,250	27.37 (26.19)	0.890	-14,250	11,375
Secondary or university studies	29 (21.75)				29.62 (26.79)			
Employment status								
Employee	38.75 (22.69)				35.5 (26.38)			
Unemployed	27.20 (18.75)	<0.001	-20,056	50,806	31.25 (20.45)	0.240	-20,056	50,806
TWD	23 (13.12)				25.25 (18.31)			
Retired	34.50 (31.62)				43.75 (28.93)			

TWD: temporary work disability.

**Table 3:** Correlation between SF-36 physical health score and SF-36 mental health score and age, number of persistent symptoms, cognitive and physical functioning, affective state, sleep quality, social support, number of steps walked, and personal construct (self-efficacy, patient´s activation and health literacy).

Variables		SF-36 Physical health			SF-36 mental health			
	Spearman Rho coefficient	P-value	Confidence interval 95%		Spearman Rho coefficient	P-value	Confidence interval 95%	
			Inferior	Superior			Inferior	Superior
Age	-0.072	0.477	-0.255	0.128	-0.064	0.525	-0.240	0.161
Number of persistent symptoms	-0.378	<0.001	-0.644	-0.297	-0.486	<0.001	-0.557	-0.130
Montreal Cognitive Assessment	0.304	0.002	0.152	0.477	0.229	0.022	0.66	0.441
Sit to Stand Test	0.524	<0.001	0.372	0.648	0.447	<0.001	0.289	0.590
Affective state (HADS)	-0.472	<0.001	-0.797	-0.563	-0.723	<0.001	-0.797	-0.615
Insomnia Severity Index	-0.430	0.097	-0.577	-0.262	-0.375	<0.001	-0.557	-0.227
Social support (MOS-SS)	0.124	0.221	-0.065	0.362	0.068	0.504	-0.133	0–334
IPAQ-SF	0.139	0.168	-0.046	0.322	0.203	0.042	-0.11	0.377
Self-efficacy	0.182	0.070	0.012	0.405	0.262	0.008	0.67	0.440
Patient´s activation	0.202	0.044	0.044	0.403	0.183	0.068	0.000	0.389
Health literacy	-0.208	0.038	-0.360	-0.003	0.182	0.182	0.065	0.190

HADS: Hospital Anxiety and Depression Scale, MOS-SS: Medical Outcomes Study Social Support Survey (MOS-SS), IPAQ-SF: International Physical Activity Questionnaire-Short Form

Regarding the linear regression model, the results are shown in Table 4, where it can be observed that the number of persistent symptoms (b = -0.900, 95% CI = [-1.523,-0.263], p = 0.008), physical functioning (b = 1.587, 95% CI = [0.679,2.521], p = 0.002) and sleep quality (b = -0.538, 95% CI = [-1.092,-0.022], p = 0.035) are predictors of SF-36 physical health score. Higher number of persistent symptoms, worse physical functioning and quality of sleep are predictors of worse quality of life, physical subscale. Whereas educational level (b = 13.167, 95% CI = [-1.391,23.535], p = 0.017), number of persistent symptoms (b = -0.621, 95% CI = [-1.245,-0.052], p = 0.057) and affective state (b = -1.402, 95% CI = [-1.964,-0.958], p<0.001) are predictors of SF-36 mental health score. Higher educational level, higher affective affectation and lower number of persistent symptoms are predictor of worse quality of life, mental subscale. The models explain 51.9% of the SF-36 physical health variance, and 54.50% of the SF-36 mental health variance.

**Table 4:** Linear regression models in relation to the SF-36 physical and mental health score.

**SF-36 Physical health score**	**Coefficient**	**P-value**	**Confidence interval 95%**		**Collinearity statistics**	
			**Inferior**	**Superior**	**Tolerance**	**VIF**
Constant	20.713	0.449	-38.564	74.170		
Number of persistent symptoms	-0.900	0,008	-1.523	-0.263	0.640	1.562
Sit to Stand Test	1.587	0,002	0.679	2.521	0.615	1.627
Insomnia Severity Index (ISI)	-0.538	0,035	-1.092	-0.022	0.554	1.807
R2	0.519					
R2adj	0.437					
**SF-36 mental health score**	**Coefficient**	**P-value**	**Confidence interval 95%**		**Collinearity statistics**	
			**Inferior**	**Superior**	**Tolerance**	**VIF**
constant	23.952	0,462	-31.904	102.756		
Educational level	13.167	0.017	1.391	23.535	0.777	1.286
Number of persistent symptoms	-0.621	0.057	-1.245	-0.052	0.689	1.451
Affective state (HADS)	-1.402	<0.001	-1.964	-0.958	0.473	2.116
R2	0.545					
R2adj	0.468					

## Discussion

This is an analysis of secondary data from an RCT conducted in Spain with 100 Long COVID patients with a diagnostic test for COVID-19 performed 12 weeks or more ago and regularly attended by primary helth care. In this way, it has been tried to obtain scientific evidence that helps to characterize clinical picture of Long COVID patients, as well as to identify factors associated with quality of life.

After becoming infected and the subsequent development of the disease, it has been determined that Long COVID patients have low levels of quality of life. Our study has identified low levels in all the dimensions evaluated in the SF-36 questionnaire, although with great variability, as expressed by the wide interquartile ranges (IQR), especially in the dimensions of physical and social function. In other words, even though the median of the eight subscales is low, there are large differences between the baseline status of patients with lower and higher scores. This could express variability of profiles, making it difficult to identify the effects of the disease. Greater variability is observed in mental health than in physical health of Long COVID patients. Along the same lines as our results, there are several studies that have determined a reduction in all vital areas after COVID-19 infection as for the most part, the participants presented persistent symptoms [36, 61, 62]. Two of these studies also used the SF-36 scale. However, other studies have been identified in which this questionnaire is used months after COVID-19 infection, which show a reduction in some areas, but not in all, since not all patients had persistent symptoms [28, 63–65]. The bibliographic review carried out with Ceban et al. (2022) verified that Long COVID patients have suffered significant functional deterioration or reduction in at least one dimension of their quality of life, compared to uninfected controls or their own state prior to infection [66]. In addition, our study verifies that women have significantly higher general health, according to the SF-36 subscale, than men. These results would be contradictory to previous evidence that has shown that women have a greater potential to develop persistent symptoms with greater intensity and repercussion than men, so their general health becomes more affected [67, 68]. However, after regrouping the eight dimensions in physical health and mental health, it is found that there are no significant differences between men and women for these two general dimensions. Therefore, it is essential to treat this data with caution and continue investigating whether there are differences by gender.

The results of this study have been able to describe a representative profile of the Long COVID patient. Our sample is made up mostly of women. This is due because the impact of this disease on women is considerably greater [69, 70]. The answer to this reality could revolve around existing immunological differences based on sex, influenced by genetic or hormonal levels among others, which contribute to women developing stronger immune responses than men, such as a greater initial inflammatory reaction and increased production of antibodies [71, 72]. Furthermore, various studies affirm that this has also happened with COVID-19, which may favor persistent symptoms [73–75]. Therefore, from a genetic perspective, sex could be playing a determining role in the development of persistent symptoms after COVID-19 infection [8, 27]. On the other hand, the average age of the participants was 48, like other studies with Long COVID patients [28, 76–78]. However, there seems to be variability in this aspect, as there are other studies in which the average age is around 10 years older [79–82]. The explanation of these results of sex and age could be justified by a greater number of women workers in social and health services in the PHC, thus considering the workers of these services as people at high risk of infection. Consequently, a notable percentage of health workers were infected during the first months of the pandemic, and they could be the future Long COVID patients [83, 84].

Regarding some socio-demographic variables that make up the Long COVID patient profile, our linear regression model has identified that a higher educational level is a predictor of worse mental health in the SF-36. Possibly, Long COVID patients with a higher educational level know about the lack of available treatments and the ignorance of health professionals. This uncertainty causes frustration among Long COVID patients, thus reducing their mental health, as a previous qualitative study indicated [29]. On the other hand, our correlation analyzes reveals that people who are actively employed or retired have a significantly higher physical health score compared to those who are unemployed or in a situation of TWD. Employment habits have a positive impact on the physical health of the population, being the day being regulated activity at work [85], recently verified by studies carried out in times of COVID-19 [86, 87]. As for retired people, it is possible that they relate some health problems to their age or inability to work. This can reduce attributing certain symptoms, such as muscle or joint pain or memory loss, to the direct effects of their illness, in addition to feeling well physically because of their long-term progressive deterioration and life trajectory.

Also, our bivariant analysis conclude that the number of persistent symptoms has a negative impact on their health i.e. the higher the number, the worse physical and mental health data. The patients in this study present 16.5 symptoms on average, with an intensity of 7-8/10. The most frequent persistent symptoms are tiredness and fatigue (98%), short attention span and concentration (89%) and myalgia (85%). These results seem to be common among those affected, being in the same line as previous evidence [70, 88]. Existing literature reinforces that patients with a greater number of persistent symptoms suffer greater repercussions on physical function and a psychological burden that generates greater emotional discomfort [89–91]. Our linear regression results also conclude that a However, our linear regression results also conclude that a higher number of symptoms would be a predictor or poor physical health, but a lower number of symptoms would be a predictor of poor mental health. This fact could be reinforced by the frequent fluctuation and scarce disappearance of the symptoms themselves. There are periods of time in which these patients may present fewer symptoms, but without recovering, which supposes an inexhaustible mental battle [92]. This reality was also reflected in another previous study, that is, it may be independent of the number of persistent symptoms of emotional well-being of the Long COVID patient [29]. In short, these results should be interpreted with caution, since the emotional well-being of these patients can be determined by other factors, such as a mental background prior to infection.

Furthermore, the results of the MoCA questionnaire verify the cognitive impairment suffered by these patients. Among the most persistent self-reported symptoms are: Short attention and concentration span (89%), Memory loss (81%) and Confusion or brain fog (71%), accompanied by neurological symptoms. These symptoms make it difficult or impossible to carry out routine activities of life, from cooking to driving, and therefore diminishing their quality of life [18, 76, 93, 94]. The study by Rass et al. (2021) used this same questionnaire 3 months after infection, finding frequent cognitive deficits, regardless of the severity of the disease, even in patients with mild disease [64]. The prevalence of this deterioration is higher after a follow-up period of 6 months, compared to other similar infections [95], which is why it is explained that in a longer time since the contagion these types of deficits persist. Thus, this cognitive impairment has the potential to affect routine actions, self-care and activities in society, which affects their quality of life [92]. Similarly, it occurs with the score obtained in the HADS questionnaire, which suggests the existence of moderate-severe anxiety-depressive disorders. This result is related to worse mental health, according to the linear regression results. Symptoms of anxiety, depression or sleep disorders would be very frequent among Long COVID patients [96, 97]. These symptoms are negative for the quality of life of people with persistent symptoms [64], as has been said.

Our results have also established a significant correlation between worse health literacy (HLS-EUQ16) and worse physical health in the SF-36. The low level of health literacy in this sample would be contradictory to the possibility of having health professionals, given their high rates of infection [98]. Research on this scale already predicts that the motivation and ability to access, understand and use information to maintain good health, which is associated with a state of good health [99, 100], so Long COVID patients would not be an exception. Also, the correlation between poor sleep quality and poor mental health should be highlighted, according to the results obtained in the ISI questionnaire and in mental health on the SF-36. Recent studies carried out with the general population affirm that sleep is causally related to the development of mental health problems [101, 102]. Reinforcing our results, several narrative reviews have verified how patients infected with COVID-19 frequently develop sleep problems accompanied by symptoms of anxiety and depression, among others [103, 104]. In addition, poor sleep quality is a predictor of impaired physical health of the SF-36, as would occur in the general population, and especially for females [105, 106].

On the other hand, correlation analyzes have concluded that physical functioning (Sit to Stand Test), self-efficacy (GSES-12) and patient activation (PAM) have the potential to promote good physical and mental health among Long COVID patients. A sedentary lifestyle contributes to the mortality of the world population, while regular and moderate physical exercise produces beneficial effects on people’s health, such as the prevention of chronic diseases and increased life expectancy [107]. Physical exercise would be a non-pharmacological strategy for the treatment of musculoskeletal-type diseases, in addition to being a stimulant of the immune system, as has been shown with pathologies similar to Post COVID-19 Syndrome [108]. For these reasons, worse physical functioning in Long COVID patients is a predictor of worse physical health in the SF-36. These results are consistent with linear regression analysis. On the other hand, the high self-efficacy of the sample would refer to self-confidence to achieve a goal. In relation to health, a health behavior such as physical exercise, persistent over time, will improve health [109–111]. In addition, the activation of patients (PAM) with chronic diseases refers to their skills, knowledge and abilities to manage their own health, as well as the health care of their environment [112, 113]. Recent studies of chronic patients relate low levels of activation with a higher degree of dependence for ADLs, worse management of their chronic conditions and progressive worsening of their symptoms [114, 115]. For this reason, despite not having identified studies that contemplate these personal constructs with Long COVID patients, they seem to be of great interest for Long COVID disease and its rehabilitation process towards a better quality of life.

### Limitations and strengths

Our study has some limitations. First, although the secondary data analysis of RCTs are a good starting point to know the baseline situations of some investigations [116], they have some limitations. For example, causal interference is not possible, and the associations can be difficult to interpret. As this was an exploratory study, no calculation of the sample size or adjustment of the p value was performed. Therefore, the findings should be interpreted with caution and should only be considered. Secondly, a convenience sampling [117] was carried out, since some people were informed through an association of those affected. However, they were asked to contact their APS physician for referral and to confirm that they met the inclusion and exclusion criteria. Thirdly, some study variables have not been included, such as reinfections/need for hospital admission or vaccination doses administered. However, it has been considered that these variables do not answer the research question of this study.

Regarding the strengths, research on the Long COVID disease is scaled up and, particularly, the impact on the quality of life of those affected. For this reason, this study adds to the existing studies that show the great affectation that these patients suffer in their quality of life, as well as the associated factors. In addition, all the participants are usually attended in PHC consultations, so our results are representative of a PHC clinical population with this pathology.

## Conclusion

In conclusion, patients diagnosed with Long COVID suffer a decline in their physical and mental health, which are proportionally and significantly correlated with the number of symptoms they present, cognitive impairment, a low affective-emotional state, related problems with their quality sleep and an acceptable level of health literacy. However, good physical functioning, as well as the patient’s personal constructs of self-efficacy and activation, can help maintain a good self-perception of physical and mental health in Long COVID patients. In addition, our linear regression analysis has identified that a greater number of symptoms, poorer physical functioning, and poorer quality of sleep are predictors of poorer physical health. Similarly, a higher educational level, a greater affective impact and a lower number of symptoms are predictors of poorer mental health. Based on the evidence generated in this study, the need to design extensive rehabilitation programs that consider both the physical and mental health of patients diagnosed with Long COVID is verified, thus obtaining an improvement in their quality of life.

## Supporting information

S1 Table Frequency and intensity of persistent symptomatology. (DOCX)
