# Supplementary material for: Clinical characterization and factors associated with quality of life in Long COVID patients: Secondary data analysis from a randomized clinical trial
Source: PLoS One. 2023 May 16;18(5):e0278728. doi: 10.1371/journal.pone.0278728 (PMC10187923; doi:10.1371/journal.pone.0278728)
Supplement: S1 Table — (DOCX) [file pone.0278728.s001.docx]

**Supporting Information**

**S1 Table. Frequency and intensity of persistent symptomatology**

| Persistent symptomatology | Total sample | |
| --- | --- | --- |
| Time since the contagious (median, IQR) | 18 (8,75) | |
| Number of persistent symptoms (median, IQR) | 16,5 (8) | |
| Persistent symptoms  Gastrointestinal symptoms  Loss of smell  Loss of taste  Blurred vision  Eye problems (dry eyes, conjunctivitis)  Tiredness or fatigue  Cough  Fever (over 38°C)  Low-grade fever (37°C - 38°C)  Chills or shivering without fever  Bruising  Myalgia  Headaches  Sore throat  Dyspnoea  Drowsiness  Dizziness  Tachycardia  Orthostatic hypotension  Joint pain  Chest pain  Back pain (cervical, dorsal, lumbar)  Neurological symptoms (tingling, spasms, etc. )  Memory loss  Confusion or brain fog  Short attention and concentration span  Loss of libido or erectile dysfunction  Altered menstrual cycle  Urinary symptoms (infections, overactive bladder)  Hair loss | Frequency %  66%  51%  48%  51%  31%  98%  49%  20%  27%  39%  22%  85%  70%  42%  60%  41%  60%  57%  20%  74%  52%  58%  59%  81%  71%  89%  46%  24%  23%  39% | Intensity (median IQR)  5 (8)  8 (5)  7 (6)  7 (3)  7 (3)  8 (3)  7 (3)  8 (2)  6 (2)  6 (3)  6 (4.25)  8 (3)  8 (3)  6 (3)  8 (2)  7 (2)  6 (3)  6 (3)  6 (4.25)  8 (3)  8 (3)  8 (3)  7 (4)  8 (3)  8 (4)  7 (3)  7 (3.5)  6 (3)  7 (3)  7 (2) |
